# The causal role of breakfast in energy balance and health: a randomized controlled trial in lean adults^1^,^2^,^3^,^4^

**DOI:** 10.3945/ajcn.114.083402

**Published:** 2014-06-04

**Authors:** James A Betts, Judith D Richardson, Enhad A Chowdhury, Geoffrey D Holman, Kostas Tsintzas, Dylan Thompson

**Affiliations:** 1 From the Departments for Health (JAB, JDR, EAC, and DT) and Biology and Biochemistry (GDH), University of Bath, Bath, United Kingdom; and the School of Life Sciences, Queen's Medical Centre, Nottingham, United Kingdom (KT).

## Abstract

**Background:** Popular beliefs that breakfast is the most important meal of the day are grounded in cross-sectional observations that link breakfast to health, the causal nature of which remains to be explored under real-life conditions.

**Objective:** The aim was to conduct a randomized controlled trial examining causal links between breakfast habits and all components of energy balance in free-living humans.

**Design:** The Bath Breakfast Project is a randomized controlled trial with repeated-measures at baseline and follow-up in a cohort in southwest England aged 21–60 y with dual-energy X-ray absorptiometry–derived fat mass indexes ≤11 kg/m^2^ in women (*n* = 21) and ≤7.5 kg/m^2^ in men (*n* = 12). Components of energy balance (resting metabolic rate, physical activity thermogenesis, energy intake) and 24-h glycemic responses were measured under free-living conditions with random allocation to daily breakfast (≥700 kcal before 1100) or extended fasting (0 kcal until 1200) for 6 wk, with baseline and follow-up measures of health markers (eg, hematology/biopsies).

**Results:** Contrary to popular belief, there was no metabolic adaptation to breakfast (eg, resting metabolic rate stable within 11 kcal/d), with limited subsequent suppression of appetite (energy intake remained 539 kcal/d greater than after fasting; 95% CI: 157, 920 kcal/d). Rather, physical activity thermogenesis was markedly higher with breakfast than with fasting (442 kcal/d; 95% CI: 34, 851 kcal/d). Body mass and adiposity did not differ between treatments at baseline or follow-up and neither did adipose tissue glucose uptake or systemic indexes of cardiovascular health. Continuously measured glycemia was more variable during the afternoon and evening with fasting than with breakfast by the final week of the intervention (CV: 3.9%; 95% CI: 0.1%, 7.8%).

**Conclusions:** Daily breakfast is causally linked to higher physical activity thermogenesis in lean adults, with greater overall dietary energy intake but no change in resting metabolism. Cardiovascular health indexes were unaffected by either of the treatments, but breakfast maintained more stable afternoon and evening glycemia than did fasting. This trial was registered at www.isrctn.org as ISRCTN31521726.

See corresponding editorial on page 503.

## INTRODUCTION

As recently identified in this and other journals, the belief that breakfast benefits health remains merely a presumption, with an outstanding need for causal data (1, 2). Epidemiology has consistently associated infrequent breakfast consumption with increased risk of adiposity, diabetes, and cardiovascular disease (3–7); yet, these findings do not infer causality because habitual breakfast consumers also tend to be nonsmokers, consume less fat and alcohol but more fiber and micronutrients, and critically, are more physically active (6, 8–10). It therefore remains to be established whether daily breakfast is a cause, an effect, or simply a marker of a healthy lifestyle.

Although much is known about the acute metabolic response over the hours after a prescribed morning meal (11–18), longer-term studies until now lacked measurement tools capable of accurately monitoring all relevant metabolic and behavioral responses in free-living humans. Moreover, previous experiments examined diets of varied meal size, composition, and/or frequency (12–24), rather than contrasting daily breakfast and extended morning fasting.

Physical activity thermogenesis is undoubtedly the most malleable component of energy expenditure, yet the few studies that specifically contrasted the relative presence or absence of regular daily breakfast consumption were either not designed to measure physical activity levels (25, 26) or were unable to detect changes in heart rate or movement/step counts extrapolated from partial daily records (8–11 h) by using wrist- or hip-worn monitors (23, 27). Such indirect estimates of energy expenditure lack reliability when applied to free-living conditions (28); neither do they provide the necessary sensitivity to detect subtle or temporal alterations in spontaneous low-to-moderate-intensity activities (29). We hypothesized that activities of precisely this nature are most responsive to modified eating patterns, and so provide novel insight by combining in-depth laboratory tests [hematology, tissue biopsies, and dual-energy X-ray absorptiometry (DXA)] with recent technological advances in continuous monitoring of physical activity thermogenesis and metabolic control in free-living humans (30).

## SUBJECTS AND METHODS

### Experimental design

The Bath Breakfast Project is a randomized controlled trial comparing the effects of daily breakfast consumption relative to extended morning fasting on energy balance and human health. This trial is registered with Current Controlled Trials (ISRCTN31521726), and the procedures followed were in accordance with the protocol approved by the National Health Service South-West 3 Research Ethics Committee (10/H0106/13). This protocol has since been published in full (30), with trial enrollment, baseline/eligibility testing, allocation, and follow-up all conducted in accordance with Consolidated Standards of Reporting Trials (CONSORT) guidelines (31). A CONSORT flow diagram (Supplemental Figure 1 under “Supplemental data” in the online issue), along with precise details of this protocol and the rationale for our approach/methods (Supplemental Figures 2 and 3 under “Supplemental data” in the online issue), are provided as online supplementary materials with this article. Here we report data for the lean cohort from the Bath Breakfast Project, classified according to DXA-derived fat mass indexes ≤11 kg/m^2^ for women and ≤7.5 kg/m^2^ for men (32), for whom recruitment and follow-up spanned dates from 10 June 2010 until 16 May 2013. These individuals all met the following inclusion criteria: aged 21–60 y, record of regular menstrual cycle/contraceptive use (if relevant), no anticipated changes in diet and/or physical activity habits during the study period, weight stable (within 1 kg over past 6 mo), non–shift workers, not pregnant or breastfeeding, and free from any other condition or behavior deemed either to pose undue personal risk or introduce bias into the experiment.

Baseline demographic and anthropometric characteristics of those who completed the trial are presented in **Table 1**. This cohort completed intensive laboratory-based assessments at baseline to determine their resting metabolic rate (via indirect calorimetry from gaseous exchange) and anthropometric characteristics, ie, DXA (Hologic Discovery W), waist and hip circumference (ie, midpoint between lowest rib and iliac crest and widest gluteal girth, respectively), and sagittal abdominal diameter (by using an abdominal caliper at the iliac crest; Holtain Limited). While participants remained in a 10-h overnight fasted state (ie, 0900 ± 1 h), a 15-mL blood sample was drawn from an antecubital vein via an indwelling cannula to determine the concentrations of key systemic metabolites/hormones via commercially available spectrophotometric assays (HDL/LDL cholesterol, triacylglycerol, nonesterified fatty acids, glucose, C-reactive protein; all from Randox Laboratories) and ELISAs [IL-6; R&D Systems; insulin; Mercodia; triiodothyronine (free-T3); ALPCO Diagnostics; thyroxine (free-T4); ALPCO Diagnostics; leptin; R&D Systems; total ghrelin and acylated ghrelin; Bertin Pharma; peptide YY and glucagon-like peptide-1; Millipore; adiponectin; R&D Systems]. A small (∼1 g) subcutaneous adipose tissue biopsy sample was then taken from the abdomen to provide estimates of tissue-specific insulin action [ie, insulin-stimulated (U-^14^C)-d-glucose uptake]. Participants then undertook an oral-glucose-tolerance test, which involved ingesting 75 g of glucose polymer in solution (Polycal; Nutricia) with 5-mL blood samples drawn every 15 min for 2 h.

**TABLE 1:** Baseline demographic and anthropometric characteristics and changes at follow-up*^1^*

	All participants (*n* = 33)		Breakfast group (*n* = 16)		Fasting group (*n* = 17)	
	Baseline	Change from baseline (95% CI)	Baseline	Change from baseline (95% CI)	Baseline	Change from baseline (95% CI)
Age (y)	36 ± 11*^2^*	—	36 ± 11	—	36 ± 11	—
Women [*n* (%)]	21 (64)	—	10 (63)	—	11 (65)	—
Frequent habitual breakfast consumer*^3^* [*n* (%)]	26 (79)	—	11 (69)	—	15 (88)	—
Anthropometric measurements						
Height (m)	1.73 ± 0.08	—	1.75 ± 0.09	—	1.71 ± 0.07	—
BMI (kg/m^2^)	22.4 ± 2.2	−0.10 (−0.21, 0.02)	22.0 ± 2.2	−0.04 (−0.24, 0.16)	22.8 ± 2.3	−0.15 (−0.27, −0.02)
DXA*^4^*						
Fat mass index (kg/m^2^)						
All	5.7 ± 2.2	−0.07 (−0.21, 0.08)	5.4 ± 2.2	−0.06 (−0.25, 0.12)	5.9 ± 2.3	−0.07 (−0.31, 0.17)
Women	6.7 ± 2.0	−0.10 (−0.30, 0.10)	6.5 ± 2.1	−0.05 (−0.30, 0.21)	6.8 ± 2.0	−0.15 (−0.49, 0.19)
Men	3.9 ± 1.3	−0.004 (−0.24, 0.23)	3.6 ± 1.0	−0.09 (−0.49, 0.31)	4.1 ± 1.6	0.08 (−0.32, 0.48)
Percentage body fat						
All	25.1 ± 8.5	−0.2 (−0.8, 0.3)	24.6 ± 8.7	−0.3 (−1.1, 0.4)	25.6 ± 8.5	−0.1 (−1.1, 0.8)
Women	29.7 ± 6.6	−0.3 (−1.1, 0.4)	29.5 ± 7.0	−0.3 (−1.2, 0.6)	29.8 ± 6.6	−0.4 (−1.7, 0.9)
Men	17.2 ± 4.7	−0.02 (−1.1, 1.0)	16.3 ± 3.5	−0.4 (−2.1, 1.3)	18.0 ± 5.8	0.4 (−1.4, 2.1)
Waist circumference (cm)	76 ± 6	−0.7 (−1.4, −0.1)*	75 ± 6	−0.9 (−1.7, −0.05)	78 ± 7	−0.6 (−1.7, 0.4)
Waist:hip ratio	0.78 ± 0.07	−0.01 (−0.02, 0.001)	0.77 ± 0.06	−0.01 (−0.02, 0.003)	0.80 ± 0.07	−0.01 (−0.02, 0.002)
Sagittal abdominal diameter (cm)	18.4 ± 1.5	−0.5 (−0.8, −0.2)*	18.3 ± 1.7	−0.5 (−1.0, −0.01)	18.6 ± 1.4	−0.5 (−0.9, −0.01)
Body mass (kg)	66.7 ± 7.9	−0.3 (−0.6, 0.02)	67.0 ± 8.3	−0.2 (−0.8, 0.4)	66.5 ± 7.8	−0.4 (−0.8, −0.1)
DXA (kg)						
Lean tissue mass*^5^*	47.1 ± 8.7	−0.08 (−0.48, 0.33)	47.7 ± 9.4	−0.08 (−0.72, 0.57)	46.4 ± 8.1	−0.08 (−0.65, 0.49)
Adipose tissue mass	16.6 ± 5.9	−0.21 (−0.63, 0.22)	16.2 ± 5.7	−0.21 (−0.76, 0.34)	16.9 ± 6.2	−0.21 (−0.91, 0.49)

1 No variable differed significantly between groups at baseline, and there were no significant treatment × time interactions. *P ≤ 0.05. DXA, dual-energy X-ray absorptiometry.

2 Mean ± SD (all such values).

3 Defined as the ingestion of ≥50 kcal within 2 h of waking on most days of the week.

4 DXA-derived fat mass index normal ranges = 5–9 kg/m^2^ (women) and 3–6 kg/m^2^ (men).

5 Lean tissue mass excludes bone mineral content.

All the above measures were followed up 6 wk later, with free-living assessments of energy intake (estimated from directly weighed food diaries) and energy expenditure (combined heart rate/accelerometry, Actiheart; CamNtech) monitored throughout the first and last week of intervention, along with continuous (5-min sampling interval) monitoring of interstitial glucose concentrations via a subcutaneous abdominal catheter (iPro; Medtronic) both to document chronic glycemic responses and to verify compliance. Eumenorrheic women provided baseline samples 2 wk before the start of the 6-wk intervention so that follow-up samples could be acquired 3–10 d after the onset of menses (ie, follicular phase). During the 6-wk intervention, participants were randomly assigned (1:1 allocation ratio) to either a group prescribed an energy intake of ≥700 kcal before 1100 daily, with at least half consumed within 2 h of waking (breakfast group) or a group that extended their overnight fast by abstaining from ingestion of energy-providing nutrients (ie, plain water only) until 1200 each day (fasting group). The randomization scheme was generated by the principal investigator (JAB) by using a computer-based random-number generator and was stratified according to baseline breakfast habits (block size = 4), with frequent breakfast consumption defined as the ingestion of ≥50 kcal within 2 h of waking on most days of the week. Investigators who enrolled participants (JDR and EAC) were unaware of these details and independently requested group assignments to prevent deciphering of the allocation sequence. Because of the self-administered nature of the treatments, it was impossible to blind participants to group allocation and neither was it possible to blind investigators for many outcomes that either required direct interaction with unblinded participants (eg, anthropometric measurements and metabolic rate) or where treatment allocation is immediately evident in the data (eg, diet records and continuous glucose monitoring). These same investigators then also shared responsibility for completing various aspects of tissue and data analysis. The intervention was applied under free-living conditions, and all other lifestyle choices were allowed to vary naturally. Compliance was confirmed via self-report and verified via continuous glucose monitoring; one participant was excluded before follow-up for noncompliance, and 4 withdrew before baseline measurements, but none attributed their withdrawal to treatment allocation (Supplemental Figure 1 under “Supplemental data” in the online issue); data reported herein are therefore only for those individuals for whom baseline and follow-up measurements were available (ie, a completers-only analysis).

### Data analysis

The primary outcome measures were a comprehensive assessment of components of energy balance under free-living conditions, which were averaged from the first and last week of intervention and therefore expressed as simple summary statistics, analyzed by using either paired or independent *t* tests for contrasts within and between groups, respectively. Secondary outcomes included regulatory/mechanistic data and markers of cardiovascular health and metabolic control at baseline and follow-up, for which treatment × time interactions were explored by using a mixed-model ANOVA with baseline breakfast habits included as a covariate. Most variables in this experiment therefore involved a single comparison between 2-level scores and so were not adjusted for multiple comparisons across the different variables reported here. However, in cases in which multiple comparisons were made within a given variable (ie, physical activity thermogenesis was partitioned according to intensity and time), a Holm-Bonferroni stepwise adjustment was applied to prevent inflation of type I error rate (33). Statistical analyses were performed by using IBM SPSS (version 21), with significance accepted at an α level of *P* ≤ 0.05. Values are means with SDs in text and with SE bars in figures, with effects expressed as change scores with 95% CIs.

## RESULTS

### Components of energy balance

#### Physical activity thermogenesis

**FIGURE 1.**
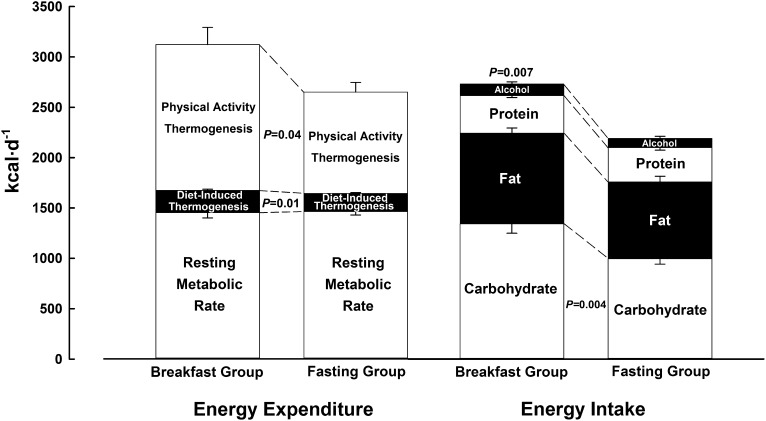
Components of energy balance under free-living conditions with either ingestion of ≥700 kcal before 1100 daily (breakfast group) or abstinence from all energy-providing nutrients until at least 1200 daily (fasting group). Values are means ± SEs. Estimated energy intake values for comparison of relative differences between groups are the average of the first [breakfast (*n* = 16) compared with fasting (*n* = 17): 2715 ± 565 compared with 2169 ± 490 kcal/d; *P* = 0.01] and last [breakfast (*n* = 16) compared with fasting (*n* = 17): 2745 ± 658 compared with 2214 ± 584 kcal/d; *P* = 0.02] week of intervention. Resting metabolic rate values (breakfast group, *n* = 16; fasting group, *n* = 16) were recorded at follow-up; diet-induced thermogenesis values (breakfast group, *n* = 16; fasting group, *n* = 17) were estimated from reported energy intake; physical activity values are the average of the first [breakfast (*n* = 15) compared with fasting (*n* = 15): 1455 ± 676 compared with 1015 ± 433 kcal/d; *P* = 0.04] and last [breakfast (*n* = 15) compared with fasting (*n* = 15): 1443 ± 705 compared with 998 ± 423 kcal/d; *P* = 0.05] week of intervention. The *P* value above the bar pertains to the overall comparison between groups; *P* values between the bars pertain to the specific comparison for the relevant component.

**FIGURE 2.**
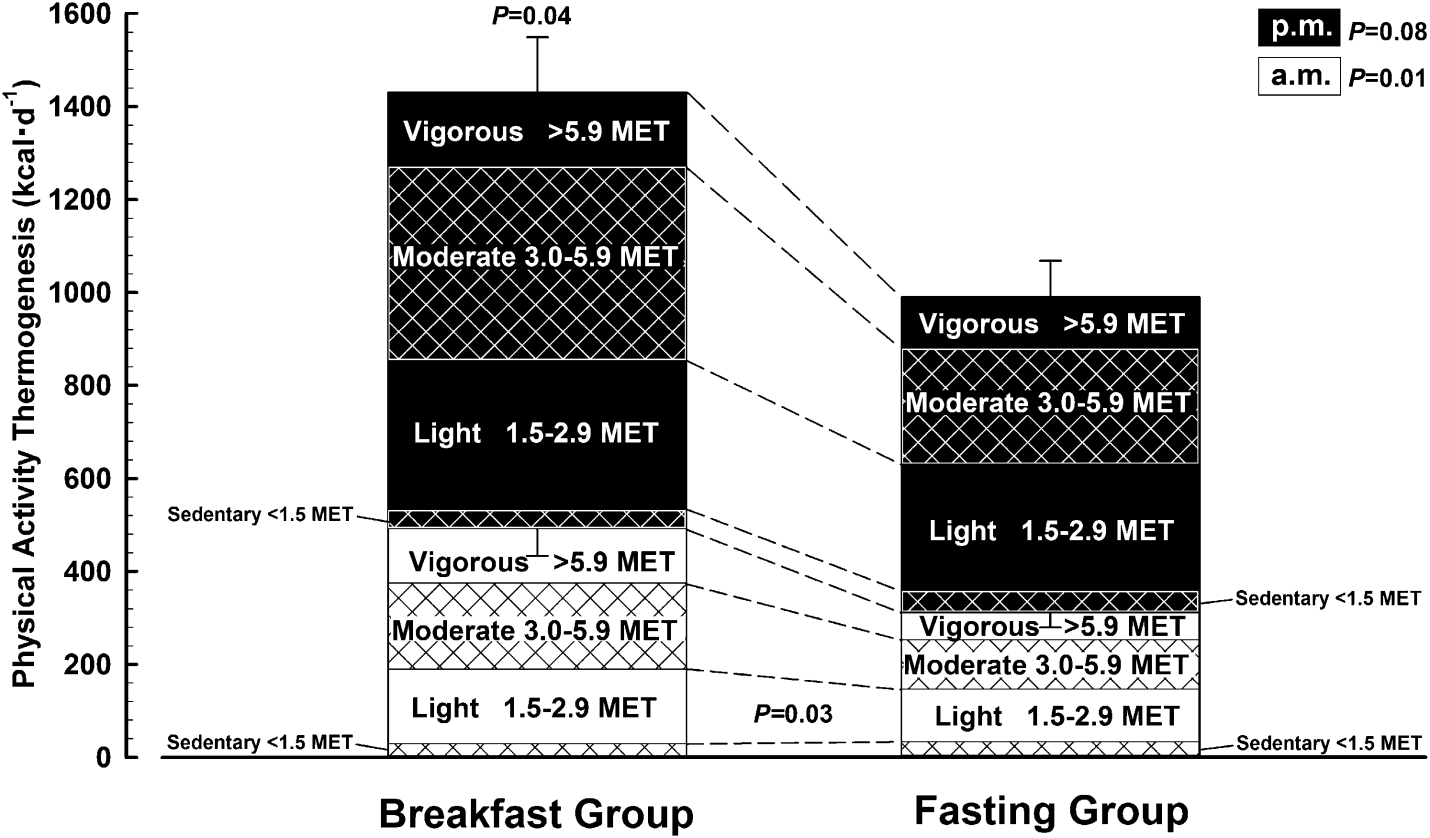
Physical activity thermogenesis under free-living conditions with either ingestion of ≥700 kcal before 1100 daily (breakfast group, *n* = 15) or abstinence from all energy-providing nutrients until at least 1200 daily (fasting group, *n* = 15). Values are means ± SEs partitioned by the time of day and intensity of energy expenditure. The *P* value above the bar pertains to the overall comparison between groups; *P* values between the bars pertain to the specific comparison for the relevant component. MET, metabolic equivalent.

The major component of energy balance that responded to treatment was physical activity thermogenesis (breakfast compared with fasting group: 1449 ± 666 compared with 1007 ± 370 kcal/d; *P* = 0.04). This overall treatment effect is apparent in **Figure 1** and partitioned in **Figure 2** to show how and when physical activity was accumulated, with a significant difference between groups before 1200 daily (breakfast compared with fasting group: 492 ± 227 compared with 311 ± 124 kcal/d; *P* = 0.01). Once classified according to accepted thresholds for the intensity of physical activities (expressed as multiples of typical resting metabolic rate, ie, a metabolic equivalent), the breakfast group consistently engaged in more “light”-intensity physical activity during the morning than did the fasting group (*P* = 0.03). There was no difference between treatment groups in daily recordings of median (range) waking times [breakfast compared with fasting group: 0727 (0625–0837) compared with 0708 (0630–0834) h] or sleeping times [breakfast compared with fasting group: 2246 (2119–2329) compared with 2247 (2132–2334) h], such that mean sleep duration was very similar between the breakfast group (483 ± 39 min/night) and the fasting group (476 ± 44 min/night).

#### Resting metabolic rate

Baseline assessments of resting metabolic rate were closely matched between the breakfast and fasting groups (ie, 1453 ± 209 compared with 1452 ± 179 kcal/d, respectively). The left side of Figure 1 presents follow-up data for these respective groups (ie, 1451 ± 210 compared with 1462 ± 146 kcal/d). Resting metabolic rate was therefore stable within 11 kcal/d from baseline to follow-up, with no difference between groups at follow-up (11 kcal/d; 95% CI: −120, 143 kcal/d).

#### Diet-induced thermogenesis

On the basis of established constants for the thermogenic effect of each macronutrient reportedly ingested according to food diaries (34), a small but consistent difference in diet-induced thermogenesis was apparent between groups (Figure 1). Specifically, the greater energy intake reported by the breakfast group led to an estimated proportionate difference in diet-induced thermogenesis (221 ± 49 kcal/d) relative to the fasting group (180 ± 39 kcal/d; *P* = 0.01).

#### Energy intake

The breakfast group reported ingesting 2730 ± 573 kcal/d relative to 2191 ± 494 kcal/d reported by the fasting group (*P* = 0.007). The right side of Figure 1 shows that the breakfast group reported ingesting most of this additional energy in the form of carbohydrate (337 ± 94 compared with 249 ± 58 g/d; *P* = 0.004), particularly in the form of sugar (149 ± 51 compared with 96 ± 38 g/d; *P* = 0.002). Analysis of the timing, type, and quantity of foods consumed at given eating occasions showed that overall daily eating patterns were unaffected by the relative consumption or omission of a daily breakfast. Specifically, once unrestricted food intake was permitted (ie, 1200 onward each day), the breakfast and fasting groups exhibited a similar frequency of consumption occasions defined as “meals” (2.2 ± 0.5 compared with 2.4 ± 0.3 meals/d; *P* = 0.2) and as “snacks” (2.1 ± 0.7 compared with 2.1 ± 0.8 snacks/d; *P* = 0.8), with no difference in the macronutrient composition of these defined eating occasions (35) between groups.

#### Energy balance regulatory hormones

Thyroid hormones that regulate resting metabolic rate were unresponsive to either of the treatments, with systemic concentrations of triiodothyronine (free-T3) and thyroxine (free-T4) closely matched between treatments at baseline and follow-up (**Table 2**). Similarly, a range of hormones implicated in the regulation of appetite and energy balance also did not differ in response between treatments. Fasted concentrations of leptin, total ghrelin, acylated ghrelin, peptide YY, glucagon-like peptide 1, and adiponectin are presented in Table 2, all of which were equivalent at baseline between treatment groups and showed no significant treatment × time interactions, such that concentrations remained similar between treatments at follow-up (although for adiponectin a significant effect of time was apparent).

**TABLE 2:** Baseline cardiovascular health, metabolic control and regulatory hormones, and changes at follow-up*^1^*

	All participants (*n* = 33)		Breakfast group (*n* = 16)		Fasting group (*n* = 17)	
	Baseline	Change from baseline (95% CI)	Baseline	Change from baseline (95% CI)	Baseline	Change from baseline (95% CI)
Cardiovascular health*^2^*						
Total cholesterol (mg/dL)	193.1 ± 34.7*^3^*	7.7 (−1.5, 15.1)	193.1 ± 46.3	3.9 (−7.7, 19.3)	193.1 ± 23.2	7.7 (−3.9, 19.3)
HDL cholesterol (mg/dL)	50.2 ± 11.6	3.9 (2.3, 7.7)*	54.1 ± 11.6	3.9 (1.2, 7.7)	50.2 ± 11.6	3.9 (1.9, 7.7)
LDL cholesterol*^4^* (mg/dL)	127.4 ± 30.9	3.9 (−3.9, 7.7)	123.6 ± 34.7	0.4 (−11.6, 11.6)	127.4 ± 23.2	3.9 (−3.9, 15.4)
Triacylglycerols (mg/dL)	75.2 ± 25.7	−3.5 (−10.6, 3.5)	76.1 ± 32.7	−0.88 (−12.4, 9.7)	74.3 ± 16.8	−5.3 (−15.9, 5.3)
NEFAs (mg/dL)	16.33 ± 7.04	−0.28 (−2.82, 2.54)	15.49 ± 5.35	1.69 (−2.25, 5.35)	17.18 ± 8.45	−1.97 (−6.20, 1.97)
IL-6 (pg/mL)	2.18 ± 1.30	−0.07 (−1.10, 0.97)	2.25 ± 1.31	−0.53 (−1.21, 0.15)	2.11 ± 1.33	0.37 (−1.63, 2.37)
CRP (mg/L)	0.65 ± 0.67	−0.11 (−0.31, 0.08)	0.79 ± 0.82	−0.13 (−0.46, 0.21)	0.53 ± 0.50	−0.10 (−0.36, 0.16)
Metabolic control						
Glucose (mg/dL)	95.5 ± 5.4	1.1 (−1.1, 3.4)	95.5 ± 5.4	1.1 (−3.6, 5.4)	97.3 ± 5.4	1.3 (−1.8, 3.6)
Insulin (μIU/mL)	3.40 ± 1.77	0.33 (−0.23, 0.90)	3.37 ± 2.15	0.35 (−0.32, 1.00)	3.43 ± 1.43	0.32 (−0.67, 1.30)
HOMA-IR*^5^*	0.81 ± 0.43	0.10 (−0.05, 0.25)	0.79 ± 0.52	0.10 (−0.06, 0.26)	0.83 ± 0.36	0.10 (−0.16, 0.36)
C-ISI Matsuda index*^5^*	11.5 ± 7.3	−0.22 (−1.66, 1.22)	12.1 ± 6.6	−0.97 (−3.70, 1.77)	11.1 ± 8.0	0.38 (−1.29, 2.05)
Index of adipose insulin sensitivity*^6^* (%)	79.1 ± 13.3	6.2 (0.2, 12.3)*	79.2 ± 12.9	9.9 (0.8, 19.0)	79.1 ± 14.1	3.3 (−5.5, 12.1)
Regulatory hormones						
Triiodothyronine (free-T3) (pg/mL)	2.90 ± 0.53	0.04 (−0.10, 0.18)	2.84 ± 0.63	0.04 (−0.20, 0.28)	2.97 ± 0.43	0.04 (−0.15, 0.23)
Thyroxine (free-T4) (ng/dL)	1.23 ± 0.16	0.04 (−0.02, 0.10)	1.24 ± 0.16	0.04 (−0.05, 0.13)	1.21 ± 0.16	0.04 (−0.05, 0.13)
Leptin (μg/L)	8.3 ± 7.5	0.4 (−1.0, 1.8)	7.8 ± 7.5	0.7 (−1.5, 2.9)	8.8 ± 7.6	0.2 (−1.9, 2.2)
Total ghrelin (pg/mL)	405 ± 163	−7 (−36, 22)	409 ± 156	13 (−33, 59)	400 ± 174	−29 (−64, 7)
Acylated ghrelin (pg/mL)	137 ± 70	5 (−2, 13)	149 ± 83	6 (−5, 18)	126 ± 55	4 (−6, 15)
Peptide YY (pg/mL)	73.8 ± 31.9	−0.5 (−7.8, 6.7)	66.5 ± 28.6	−2.8 (−12.4, 6.8)	81.1 ± 34.2	1.9 (−10.4, 14.1)
GLP-1 (pg/mL)	16.50 ± 14.19	3.96 (−1.98, 9.90)	17.49 ± 17.16	2.31 (−2.97, 7.59)	15.18 ± 10.56	5.61 (−6.27, 17.49)
Adiponectin (mg/L)	8.9 ± 3.4	0.8 (0.2, 1.5)*	9.8 ± 3.4	0.4 (−0.5, 1.3)	8.1 ± 3.4	1.3 (0.3, 2.2)

1 No variable differed significantly between groups at baseline, and there were no significant treatment × time interactions. *P ≤ 0.05. C-ISI, Composite-Insulin Sensitivity Index; CRP, C-reactive protein; GLP-1, glucagon-like peptide 1; NEFA, nonesterified fatty acid; OGTT, oral-glucose-tolerance test; T3, triiodothyronine; T4, thyroxine.

2 SI conversions: cholesterols × 0.0259 = mmol/L; triacylglycerol × 0.0113 = mmol/L; NEFA × 0.0355 = mmol/L; IL-6 × 0.131 = IU/mL; CRP × 9.524 = nmol/L; glucose × 0.0555 = mmol/L; insulin × 6.0 = pmol/L; free-T3 × 1.54 = pmol/L; free-T4 × 12.87 = pmol/L; leptin × 0.0625 = nmol/L; ghrelin × 0.296 = pmol/L; peptide YY × 4.31 = pmol/L; GLP-1 × 0.303 = pmol/L.

3 Mean ± SD (all such values).

4 Calculated by using the Friedewald equation: LDL cholesterol = total cholesterol − HDL cholesterol − (triacylglycerol/2.2).

5 HOMA-IR = [fasted insulin (μIU/mL) × fasted glucose (mmol/L)]/22.5; C-ISI Matsuda index = 10,000/√[fasted glucose (mg/dL) × fasted insulin (μIU/mL)] × [mean glucose over 120-min OGTT (mg/dL) × mean insulin over 120-min OGTT (μIU/mL)].

6 Based on insulin-stimulated adipose tissue [U-^14^C]-d-glucose uptake in cells treated with 50 pmol insulin/L expressed as a percentage of maximal/supraphysiologic stimulation with 20 nmol insulin/L [as used previously (36)].

### Health risk factors

#### Anthropometric measurements

Relative and absolute indexes of body mass and both whole-body and central adiposity did not differ between treatments at baseline or follow-up; therefore, there was no significant difference in the response over time between treatments (Table 1).

#### Cardiovascular health

The blood lipid profiles presented in Table 2 all approximate the normal healthy range for each subfraction and for C-reactive protein concentrations, with the exception of LDL cholesterol at follow-up (ie, 131.3 ± 27.0 mg/dL), which may be considered borderline high for risk of cardiovascular disease events according to established classifications (ie, >130 mg/dL) endorsed by the American Heart Association (37). However, other than a small increase in HDL cholesterol from baseline to follow-up in both groups, these variables were unresponsive to either of the treatments.

#### Metabolic control

**FIGURE 3.**
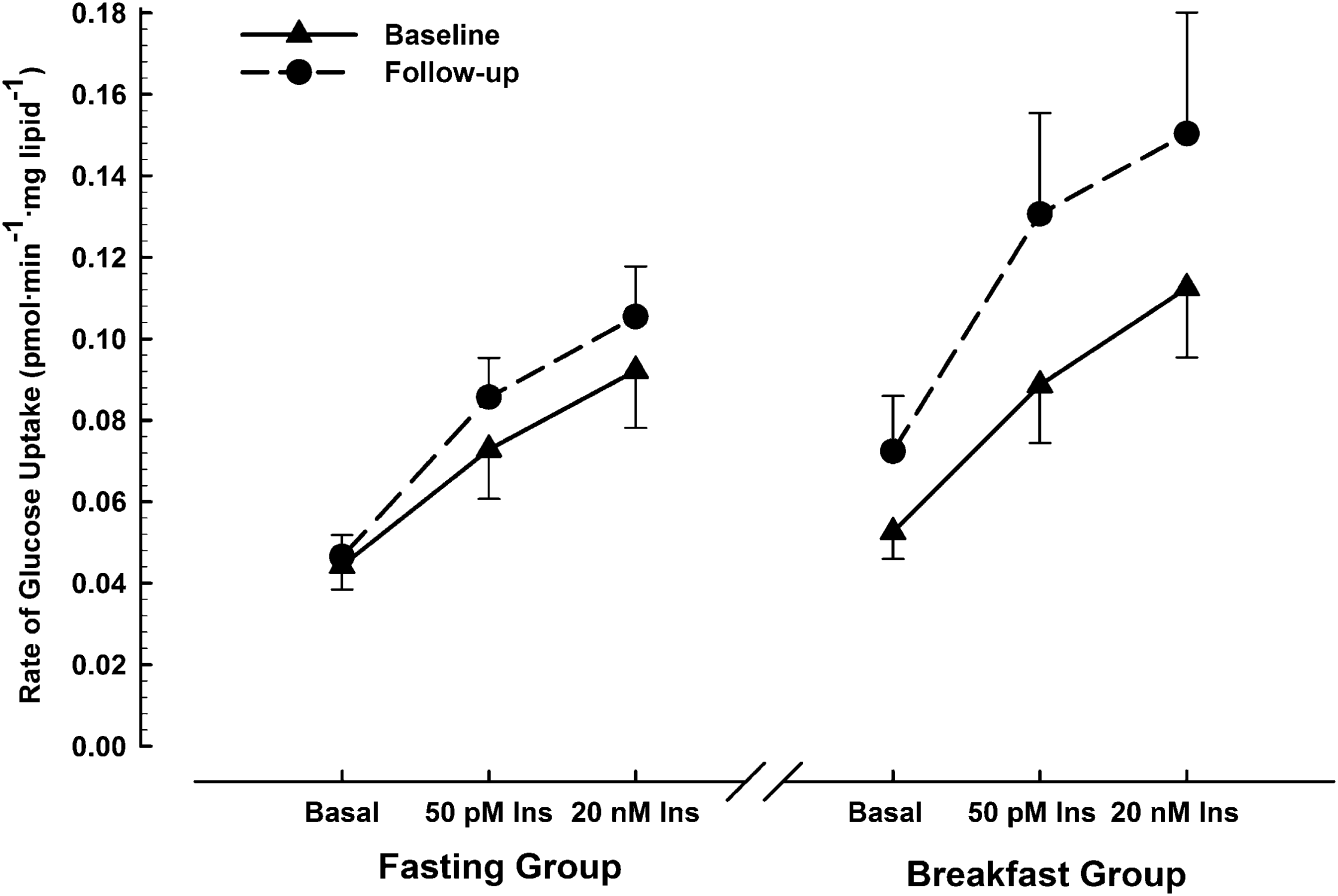
Rates of [U-^14^C]-d-glucose uptake in adipocytes under basal, physiologic (50 pmol insulin/L), and supraphysiologic (20 nmol insulin/L) conditions, measured at baseline and after 6-wk ingestion of ≥700 kcal before 1100 daily (breakfast group; *n* = 13) or abstinence from all energy-providing nutrients until at least 1200 daily (fasting group; *n* = 16). Values are means ± SEs. Three-factor ANOVA (treatment × time × insulin) showed a significant effect of treatment (*F* = 4.2, *P* = 0.05) and insulin (*F* = 17.3, *P* < 0.001) with no significant effect of time nor any interaction of these factors. Ins, insulin.

Fasted plasma glucose and serum insulin concentrations were unaffected by 6 wk of either daily breakfast or extended morning fasting (Table 2). Similarly, the HOMA-IR and Matsuda indexes did not show any impact of either of the treatments on pancreatic β cell function or whole-body insulin sensitivity. Similarly, the index of insulin sensitivity specific to adipose tissue presented in Table 2 showed no significant treatment × time interaction, nor any significant difference between groups at follow-up. This index is derived from the rates of tissue-specific glucose uptake presented in **Figure 3**.

Subcutaneous glucose was monitored continuously throughout the first and last week of intervention. Peak glucose concentrations from waking until 1200 were consistently higher with breakfast (7.6 ± 1.2 mmol/L) than with fasting (6.5 ± 1.0 mmol/L) throughout the first and last week (treatment effect: *F* = 9.6, *P* < 0.01) with no treatment × time interaction. Similarly, mean glucose concentrations during the morning did not respond differently between treatments over time but were significantly higher with breakfast (5.4 ± 0.5 mmol/L) than with fasting (5.1 ± 0.5 mmol/L) when considered across both weeks (treatment effect: *F* = 4.3, *P* = 0.05).

**FIGURE 4.**
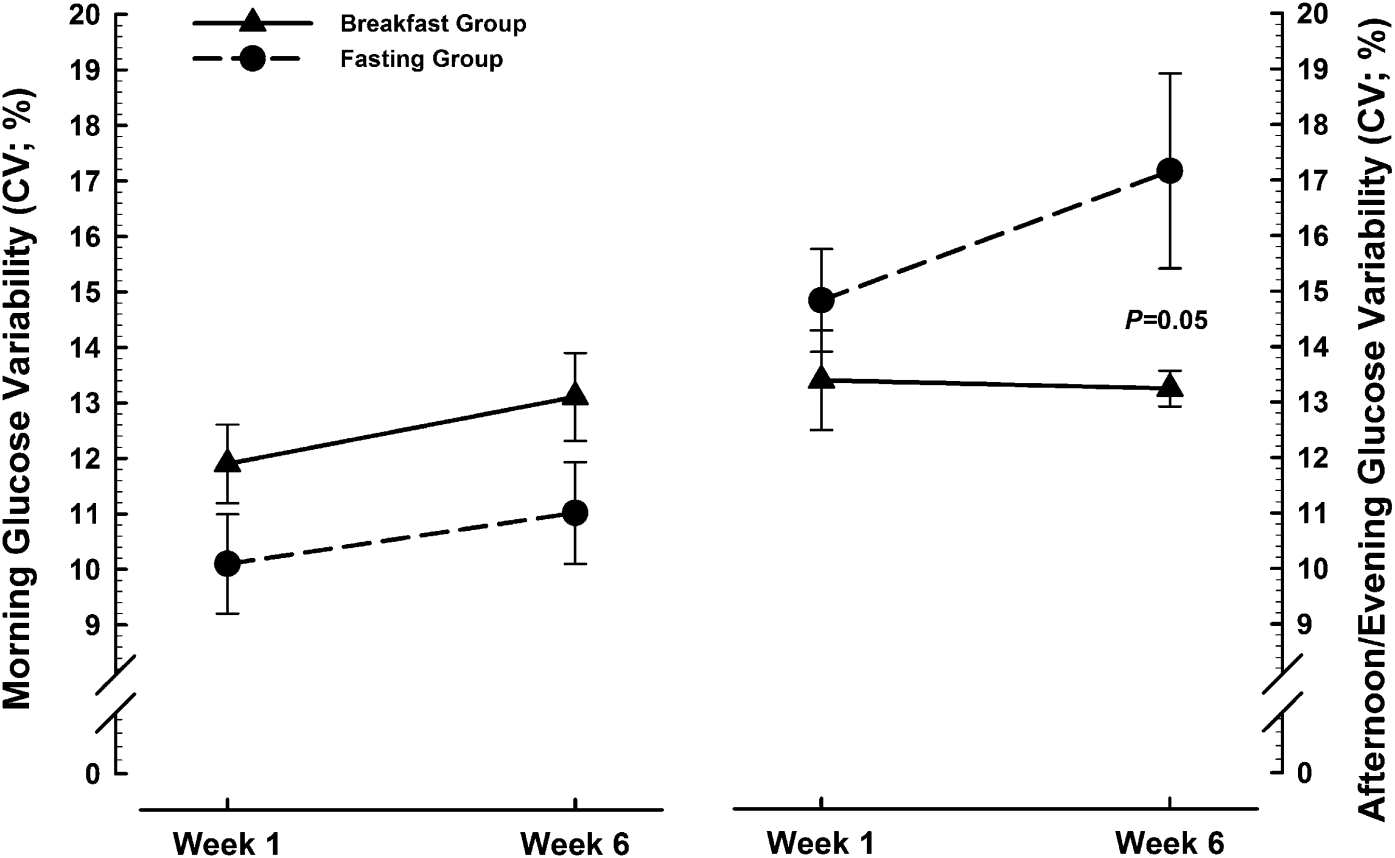
Glucose variability before and after 1200 (expressed as CVs) derived from subcutaneous glucose concentrations continuously monitored during the first and last week of either ingesting ≥700 kcal before 1100 daily (breakfast group; *n* = 16) or abstaining from all energy-providing nutrients until at least 1200 daily (fasting group; *n* = 16). Values are means ± SEs. Two-factor ANOVA (treatment × time) showed a nonsignificant trend for treatment (*F* = 3.9, *P* = 0.06) with no significant effect of time nor any interaction of these factors during the morning (left side of panel) and a significant effect of treatment (*F* = 6.2, *P* = 0.02) with nonsignificant trends for time (*F* = 3.8, *P* = 0.06) and treatment × time (*F* = 3.6, *P* = 0.07) during the afternoon/evening (right side of panel).

The CV (SD corrected for mean) is the preferred method for expressing glucose variability (accumulated hyper-/hypoglycemic episodes) when continuously monitored glucose data are available for individual patients (38). This measure is shown in **Figure 4**, both from waking until 1200 (left side) and from 1200 until sleep (right side). There was a nonsignificant trend across weeks 1 and 6 toward greater glucose variability before 1200 in the breakfast group compared with the fasting group (treatment effect: *F* = 3.9, *P* = 0.06). Conversely, the fasting group exhibited greater glucose variability across weeks 1 and 6 from 1200 onward (treatment effect: *F* = 6.2, *P* = 0.02), with a nonsignificant tendency to increase over the course of intervention (treatment × time interaction: *F* = 3.6, *P* = 0.07) such that the greatest group difference was apparent during week 6 (3.9%; 95% CI: 0.1%, 7.8%). Beyond the above effects, continuous glucose monitoring showed similar peak, mean, and variability values between groups during sleep, from 1200 until sleep, and over the full 24-h period.

## DISCUSSION

Here we provide the first report, to our knowledge, of all components of energy balance with regular daily breakfast relative to extended morning fasting measured under otherwise free-living conditions. Contrary to popular belief, there was no metabolic adaptation (ie, increased resting metabolism) to 6 wk of daily breakfast nor any meaningful suppression of energy intake later in the day. Rather, the major factor that compensated for the prescribed difference in morning eating patterns was that regular daily breakfast resulted in significantly higher physical activity thermogenesis than occurred with extended morning fasting. This difference in the energy expended via daily physical activities was partially attributable to a significant difference in light-intensity activities performed during the morning and has never previously been objectively measured. Although fasting until 1200 every day for 6 wk presented no negative cardiovascular effects as reflected by the concentration of blood metabolites under fed and fasted conditions, there was a 10% increase in adipose tissue insulin sensitivity within the breakfast group. Conversely, those in the fasting group exhibited no such effect on adipose tissue insulin sensitivity (although this did not significantly differ from the breakfast group at follow-up) but instead experienced progressively more variable interstitial glucose concentrations during the afternoon/evening, with significantly less tightly regulated glucose control than the breakfast group by the final week of the intervention. Further research is required to establish whether similar responses would be apparent in more overweight, less healthy, and/or less physically active individuals.

Because physical activity thermogenesis differed significantly between treatments in light-intensity activities, this suggests that breakfast may have affected spontaneous behaviors as opposed to conscious decisions to participate in structured physical activity or exercise. This reasoning may explain why attempts to explore the causal effects of breakfast on energy expenditure by using physical activity records/diaries would not be sensitive to such an effect. Nonetheless, cross-sectional studies have detected slightly higher self-reported overall physical activity levels among breakfast eaters (39), and several recent cross-sectional studies using accelerometry reported correlations between habitual breakfast consumption and physical activity levels, particularly during the morning (40, 41). The present study therefore shows for the first time to our knowledge that these interesting correlations do indeed have a causal component. In addition, the present study shows the value of combined heart rate/accelerometry in revealing not only total 24-h physical activity thermogenic responses but also temporal changes in physical activity patterns (ie, when and how overall energy expenditure is accumulated).

The overall difference in physical activity thermogenesis between the breakfast and fasting groups reported in this study (Figure 2) was equally apparent whether considering the first (1455 ± 676 compared with 1015 ± 433 kcal/d; *P* = 0.04) or last (1443 ± 705 compared with 998 ± 423 kcal/d; *P* = 0.05) week of intervention. This indicates that the effect of morning eating patterns on physical activity levels may be direct and immediate rather than secondary to accumulated physiologic adaptations with sustained exposure to the presence or absence of daily breakfast. One possible immediate mechanism is that an increase in glucose availability may directly signal the transition from the fasted to fed state and thus enable nonessential physical activity energy expenditure above basal levels (40). Despite the widespread belief that an extended period of fasting and/or hypoglycemia can result in lethargy, this hypothesis has never been empirically tested. Transient periods of reactive hypoglycemia (≤3.3 mmol/L) between meals certainly do not explain the general fatigue often attributed to “low blood sugar” (43), yet no previous study has examined the effects of a more prolonged but less pronounced period of reduced glucose availability on physical activity levels in humans. The present study therefore provides tentative evidence that skipping a morning meal altogether and thus delaying the transition to fed-state glucose availability may impose a limit on the amount of energy expended via physical activities.

Overall reported dietary energy intake was 539 kcal/d lower when fasting until 1200 than when consuming a breakfast of ≥700 kcal before 1100 daily, with no difference between treatments in terms of the frequency, timing, or composition of meals consumed from 1200 onward. This reflects minimal dietary compensation for the energy deficit imposed by morning fasting but also indicates that the treatment effect for afternoon/evening glucose variability is not attributable to group differences in eating patterns during that period. Although it could be contended that the higher afternoon/evening glucose variability in the fasting group might simply reflect an initial transition from a lower starting (ie, still fasted) value, this effect would be apparent in the first week as well as in the last. The group difference in afternoon-evening glucose variability may instead therefore be explained by the Staub-Traugott (or “second meal”) effect, which describes how an initial meal can reduce the acute glycemic response to subsequent feedings within hours of the first (44). The present findings suggest that regularly skipping breakfast may elicit adaptations to progressively increase systemic glucose appearance and/or impair glucose disposal when the overnight fast remains unbroken until after 1200.

On the basis of the distinct free-living responses of the treatment groups reported here, it would clearly be informative to have data characterizing participants’ habitual lifestyles in the absence of intervention (particularly in terms of diet and physical activity habits). Given the already intensive nature of this trial [including measures beyond those reported here (30)], additional prolonged lifestyle monitoring was not deemed feasible because this could negatively affect recruitment/attrition rates while also increasing the risk of measurement fatigue. However, our main finding with regard to physical activity thermogenesis is most likely a genuine response to treatment because of the following 3 key interrelated reasons: *1*) it is highly unlikely that randomization of a group this size would generate such a marked and consistent difference in this variable (442 kcal/d; 95% CI: 34, 851 kcal/d); *2*) this difference was most consistently observed during the morning, thus coincident with the time of day at which treatments were applied; and *3*) all of the other laboratory-based variables that could be measured acutely before intervention were equivalent between groups (ie, resting metabolic rate and all other variables presented in Tables 1 and 2).

This study was designed to examine mechanisms linking daily breakfast and components of energy balance in free-living humans and not the long-term impact of breakfast habits on weight change. Therefore, whereas we detected no significant difference in weight loss between treatments (Table 1), longer-term clinical trials are needed to examine the effects of continued exposure to extended morning fasting. However, what can be confidently concluded from the current data is that daily morning fasting clearly did not cause weight gain in this population, as might be hypothesized in view of epidemiologic evidence showing positive associations between regularly skipping breakfast and weight gain/status (3–6).

We conclude that daily breakfast is causally linked to higher physical activity thermogenesis in lean adults, with limited evidence of any dietary compensation later in the day nor any change in resting metabolism. Blood metabolites indicative of cardiovascular health and metabolic control were not significantly affected by breakfast or fasting and neither was there any difference in adipose tissue glucose uptake between these treatments. However, regular daily breakfast maintained more stable glucose responses during the afternoon and evening.

## Supplementary Material

Supplemental data
