# Supplementary material for: The causal role of breakfast in energy balance and health: a randomized controlled trial in lean adults1
Source: Am J Clin Nutr. 2014 Jun 4;100(2):539–47. doi: 10.3945/ajcn.114.083402 (PMC4095658; doi:10.3945/ajcn.114.083402)
Supplement: Supplemental data [file supp_100_2_539__index.html]

The causal role of breakfast in energy balance and health: a randomized controlled trial in lean adults — Supplemental data 

# The causal role of breakfast in energy balance and health: a randomized controlled trial in lean adults

## Supplemental data

**Files in this Data Supplement:**

- Supplemental data - Figure 1
- Supplemental data - Text and Figures 2 and 3
