# Supplementary material for: The causal role of breakfast in energy balance and health: a randomized controlled trial in lean adults1
Source: Am J Clin Nutr. 2014 Jun 4;100(2):539–47. doi: 10.3945/ajcn.114.083402 (PMC4095658; doi:10.3945/ajcn.114.083402)
Supplement: Supplemental data [file 114.083402_ajcn083402SupplementaryData1.docx]

**Supplemental Figure 1:** Flow diagram illustrating progress through each phase of the trial. NB Consistent with the published protocol for this trial ([1](#_ENREF_1)), lifestyle maintenance/monitoring was applied to any willing participant eligible for randomization into the breakfast/fasting treatment groups (thus broadly equivalent to the main study population) but unable to commit to the main study for other reasons (e.g. impossible to schedule trials in the required time-frame, unwilling to provide tissue samples, *etc*.). Monitoring free-living responses in the absence of any intervention therefore provides context regarding the extent to which those free-living measures alone (i.e. dietary and physical activity monitoring) may impact typical energy intake (2391 ± 626 kcal·d^-1^), physical activity levels (1230 ± 603 kcal·d^-1^) and change in body mass body mass over 6 weeks (-0.09 kg; 95% CI=-0.8, 0.6) under conditions where diet and physical activity are monitored as applied in the main experiment.

1. Betts JA, Thompson D, Richardson JD, et al. Bath Breakfast Project (BBP) - Examining the role of extended daily fasting in human energy balance and associated health outcomes: study protocol for a randomised controlled trial [ISRCTN31521726]. Trials 2011;12:172.
