# Supplementary material for: The causal role of breakfast in energy balance and health: a randomized controlled trial in lean adults1
Source: Am J Clin Nutr. 2014 Jun 4;100(2):539–47. doi: 10.3945/ajcn.114.083402 (PMC4095658; doi:10.3945/ajcn.114.083402)
Supplement: Supplemental data [file 114.083402_ajcn083402SupplementaryData2.doc]

*Online Supplementary Material - Full methodology consistent with the trial protocol published at:*

Betts J. A., Thompson D., Richardson J. D., Chowdhury E. A., Jeans M., Holman G. D. & Tsintzas K. (2011) Bath Breakfast Project (BBP): Examining the role of extended daily fasting in human energy balance and associated health outcomes: study protocol for a randomized controlled trial [ISRCTN31521726] *Trials* 12: 172.

#

## METHODS/DESIGN

Approach to the Research Question

As illustrated in **Supplemental Figure 2**, extended daily fasting has the potential to impact upon adiposity, insulin resistance and cardiovascular disease via the acute influences of a single extended daily fast on a given day in terms of contributing to a more positive daily energy balance (***Objective i***) but also via the chronic metabolic and/or behavioural adaptations which may occur with habitual exposure to extended daily fasting over a more prolonged period (***Objective ii***). This project will take advantage of recent technological advances to comprehensively assess all aspects of energy balance and the physiological mechanisms which may underpin causal relationships between feeding frequency and energy balance (i.e. energy balance hormones). Furthermore, this approach of applying state-of-the-art analytical techniques to further progress current understanding will also provide valuable insight by examining selected health-related outcomes at a variety of levels ranging from molecular to whole-body (***Objective iii)***. Specifically, many components and consequences of positive energy balance (e.g. poor dietary composition, sedentary behaviour and adiposity) have been well established as independent risk factors for insulin resistance and associated cardiovascular disease,[^1-3^](#_ENREF_1) with chronic low-grade inflammation strongly implicated in the latter.[^4^](#_ENREF_4) In particular, systemic concentrations of C-reactive protein (CRP) have been shown to exhibit positive correlations with fasted glucose concentrations,[^5^](#_ENREF_5) atherosclerotic progression[^6^](#_ENREF_6) and the incidence of initial coronary heart disease events.[^7^](#_ENREF_7) It is therefore anticipated that the range of measures described below will address the stated objectives both from a basic and an applied science perspective to establish causal mechanisms and relationships to clinical outcomes, thus providing detailed yet practically valuable understanding in relation to public health policy and clinical practice.


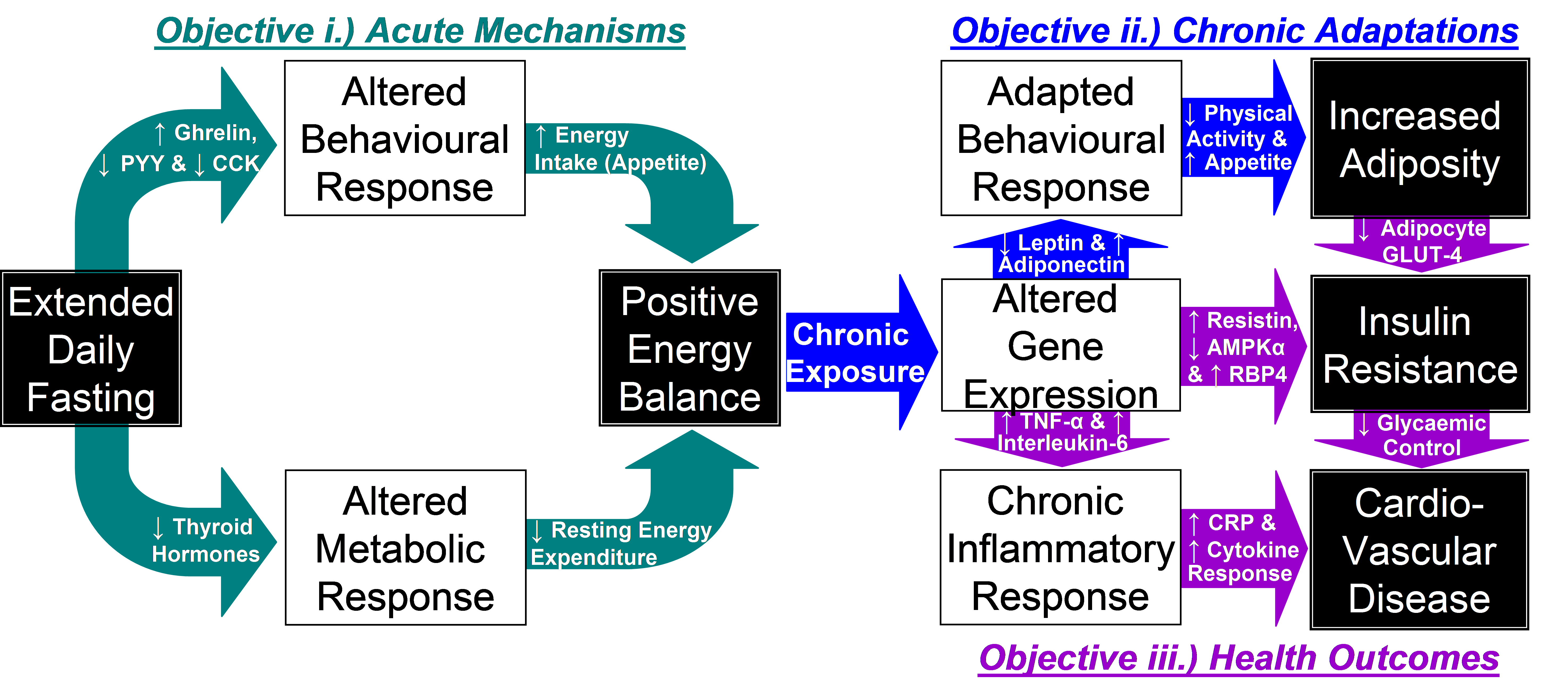


**Supplemental Figure 2:** Proposed mechanistic pathway underlying the relationships between extended daily fasting, energy balance and health outcomes.

Trial Design

**Supplemental Figure 3** illustrates the planned course of progress through this trial consistent with current CONSORT guidelines for reporting randomized trials.

**Supplemental figure 3:** Flow diagram illustrating progress through each phase of the trial [adapted from current CONSORT guidelines[^8^](#_ENREF_8)].

Participants/Eligibility

A total cohort of 60-70 men and women will take part in the main part of the experiment, half of whom will be broadly classified as normal weight (BMI ~20-25 kg∙m^-2^), whereas the other half will be obese (BMI ≥30 kg∙m^-2^). This broad classification according to BMI is intended to generate two separate and diverse overall populations for subsequent more accurate and gender specific stratification based upon DEXA-derived fat-mass index (♂ FMI ≤7.5 kg∙m^-2^<; ♀ FMI ≤11 kg∙m^-2^<), thus controlling for differences in lean tissue[^9^](#_ENREF_9) and allowing separate analyses according to whether a given individual is ordinarily able to attain long-term energy balance. Furthermore, baseline breakfast habits will be included as a co-variate in our analysis to consider the distinct responses of frequent *versus* infrequent breakfast consumers, with frequent breakfast consumption defined as the ingestion of ≥209 kJ within two hours of waking on most days of the week. Both the above factors will be included in a stratified randomization scheme, with generation of 4 separate block randomization schedules to ensure an equal distribution of normal/overweight and in/frequent breakfast consumers between treatment groups. These randomization schedules will be produced by the principal investigator using a computer-based random number generator, with full details of the overall randomization scheme only to be published in full once group allocation is complete to complicate deciphering of the allocation sequence by those involved in trial enrolment.[^10^](#_ENREF_10) Further procedures to conceal the allocation sequence are that the two individuals responsible for trial enrolment (JR & EC) will independently request group assignments from the principal investigator via email immediately upon verification of eligibility according to pre-stated inclusion criteria. Combining these separate requests into the same set of randomization schedules in order of receipt therefore renders it impossible for either individual to confidently predict upcoming group assignments without prior knowledge of the other’s requests. Moreover, even with this knowledge, randomization at the point of objectively establishing eligibility ensures that the sequence in which requests are received is determined before adequate information becomes available to predict the likely responsiveness of any given individual to each treatment.

Lastly, any volunteer fulfilling all eligibility criteria (thus equivalent to the main study population) but unable to commit to the main study for other reasons (e.g. impossible to schedule trials in the required time-frame, unwilling to provide tissue samples, etc.) will be invited to participate as part of a ‘true’ control group. This group will complete only the free-living element of the study (Phase II; see later) while simply maintaining their usual lifestyle, therefore providing some background context regarding the extent to which the free-living measures themselves (i.e. dietary & physical activity monitoring) may impact outcomes but without necessitating any extended visits to the laboratory for tissue samples. These volunteers for the proposed work will be recruited from the local community (South West UK) via public advertisement (e.g. local press/radio) and will not be provided any financial incentive for participation. The protocol described herein was reviewed and approved by the National Health Service South West 3 Research Ethics Committee (10/H0106/13).

*Inclusion Criteria*

- Aged 21-60
- Body Mass Index 20-25 kg∙m^-2^ or >30 kg∙m^-2^
- Able and willing to safely comply with all study procedures
- Able to provide written informed consent for participation
- Females must maintain a record of regular menstrual cycle phase or contraceptive use
- No anticipated changes in diet and/or physical activity habits during the study period (e.g. pre-planned holidays, diets/exercise plan, *etc*.)

*Exclusion Criteria*

- Any reported condition or behaviour deemed either to pose undue personal risk to the participant or introduce bias into the experiment
- Any reported use of substances which may pose undue personal risk to participants or introduce bias into the experiment
- Any individual whose habitual lifestyle does not conform to a standard sleep-wake cycle (e.g. shift workers)
- Simultaneous or recent (i.e. last 3 months) participation in another clinical trial or blood donation, to allow full recovery of blood volume[^11^](#_ENREF_11)
- Any reported recent (i.e. last 6 months) shift (>1 kg) in body mass
- Any reported tendency towards keloid scarring
- Any reported bleeding disorder
- Females known to be pregnant or planning to become so over the course of the study
- Females with oral or implanted contraceptives fitted within 6 months of participation[^12^](#_ENREF_12)
- Females who are breastfeeding

Statistical Approach/Power Estimation

The number of research participants to be recruited was estimated based upon a worthwhile effect dictated by the smallest shift in energy balance necessary to induce chronic weight loss. Specifically, one of the most similar studies to the proposed work applied doubly-labelled water to measure average daily energy expenditure in a population not dissimilar to that planned,[^13^](#_ENREF_13) from which a mean of ~12000 kJ∙d^-1^ might be expected with a standard deviation in the region of ~2000 kJ∙d^-1^. The daily breakfast to be consumed in this trial will provide in excess of ~3000 kJ∙d^-1^ and our pilot work using this breakfast indicates that this is likely to stimulate metabolism in the lead up to lunch by ~150 kJ∙d^-1^ and reduce energy intake at lunch by ~150 kJ∙d^-1^, relative to continued fasting. Therefore, for breakfast consumption to exert a worthwhile effect on net energy balance (i.e. sufficient to compensate for the energy it provides directly), physical activity energy expenditure would need to increase and/or subsequent energy intake to decrease by a further 2700 kJ∙d^-1^. Using the above figures, a worthwhile increase in energy expenditure would require ~14 participants in each treatment group to confer a 90% probability of detecting such an effect statistically using a two-tailed *t*-test with an alpha level of 0.05. A total of 70 participants will therefore be recruited for the main study treatment arms in view of the 14% drop-out rate reported by Schlundt *et al*. (1992) when placing similar demands on volunteers,[^14^](#_ENREF_14) thus aiming to achieve at least 60 participants to provide sufficient statistical power even when the overall breakfast *versus* no breakfast groups are stratified according to FMI (with baseline breakfast habits, gender and age included as co-variates in the analysis). Nonetheless, to minimise the negative impact of loss to follow-up on validity, participants will be advised at trial enrolment to very carefully consider the required investment of time and effort relative to their current and forthcoming commitments outside of the trial, such that exclusions based on likely withdrawal can be made prior to randomization.[^15^](#_ENREF_15) Any unforeseen circumstances or withdrawal of consent subsequently resulting in loss to follow-up will be documented fully in the final trial report. Data collected from these individuals to the point of loss will then be compared with those who complete each treatment to determine whether the overall conclusions of the trial may be generalised beyond populations reflective of the latter (i.e. to anyone willing to be randomized).

As with the primary outcome measure, secondary variables will also be analysed using either paired or independent *t*-tests applied to simple summary statistics between groups or trials, respectively (e.g. fasted/peak values, incremental area under curve, average value over 24 h, *etc*.). Given that these sample size estimations are based on a primary outcome that inherently displays a relatively large inter-individual variability, it is anticipated that the more consistent responses of secondary variables will also be detectable with the selected sample size.

Experimental Protocol

***Objective i –*** The first phase of the proposed work (Phase I) will involve two laboratory-based examinations of the acute metabolic and behavioural responses to extended fasting relative to ingestion of a standardised breakfast. These trials will be applied in a randomized and counterbalanced order with 3-28 days interval except for eumenorrheic women, whose trials will be separated by 28±2 days and only at least 3 and at most 10 days after the onset of menses (i.e. follicular phase) to ensure that the effects of menstrual cycle on the majority of hormones and therefore resting metabolic rate (RMR) and appetite will be both minimal and standardised between trials.[^16-18^](#_ENREF_16) These acute trials will be conducted in our resting metabolism laboratory in accordance with current guidelines for best practice in measuring resting metabolic rate.[^19^](#_ENREF_19) Most notably, ambient temperature in this laboratory is maintained between 20 and 25 ºC (with intra-individual trials standardised within 2 ºC) and participants will arrive for testing at 8 am (±1 h) in a 10 hour fasted state but having ingested 1 pint of plain water upon waking following standardised physical activity and diet (the latter to incorporate either daily breakfast or extended fasting according to the group randomization described later, thus permitting meaningful comparisons with follow-up visits). Repeated 5 minute resting expired gas samples will be collected over 30 minutes (after 20 minutes of quiet rest) to establish RMR and substrate selection before a cannula will be fitted to an antecubital vein for the acquisition of a baseline 15 ml blood sample, along with further samples throughout that day.

At this stage, participants will provide ratings of hunger and appetite using validated visual analogue scales,[^20^](#_ENREF_20) for subsequent follow-up post-breakfast and also pre-post lunch and at the end of day. They will then receive either no breakfast or a typical standardised breakfast (composed of cereal, toast and orange juice) with the opposite treatment applied in each participant’s second trial). The specific food and macronutrient composition of this breakfast has been described previously[^21^](#_ENREF_21) and provides quantities intended to deliver 14 mg carbohydrate per kJ of each individual’s previously established RMR. Fifteen minutes later or upon completion of the breakfast (if longer than 15 minutes is required) a 3 hour post-prandial period will commence, involving collection of all urine output (for determination of urea nitrogen excretion) with hourly expired 5 minute gas samples to assess changes in substrate metabolism (i.e. carbohydrate, lipid and protein oxidation) and DIT (i.e. post-prandial energy expenditure minus RMR). Fifteen millilitre blood samples (including 5 ml waste) will also be drawn 15, 30 & 60 minutes post-breakfast, then at hourly intervals to determine systemic concentrations of: glucose; lactate; insulin; NEFA; urea; ‘energy balance hormones’ that are known to play a central role in the regulation of metabolic rate, appetite and spontaneous physical activity (e.g. free thyroxine, adiponectin, CCK, total/acylated ghrelin, leptin & PYY); and also cytokine concentrations (e.g. interleukin-6 and TNF-α), in view of recently documented differences in post-prandial inflammatory responses to varied meals across different populations[^22^](#_ENREF_22) (with low/high density lipoprotein cholesterol and CRP only followed-up at the 3 hour time-point). Participants will then be provided with a test meal of standardised composition (1 kg cooked Sainsbury’s™ penne pasta and Ragu™ traditional tomato sauce; prepared at a ratio of 1:1 uncooked mass) along with *ad libitum* plain water (although participants will ingest the same volume of water as in their first trial during all subsequent visits), which we have previously employed during pilot work to estimate likely differences in voluntary energy intake. At this stage, participants will be left alone in the laboratory and receive a recorded message stating: “We ask that you continue eating until you have satisfied your hunger. The lunch will remain in front of your for at least 30 minutes, at which point the post-lunch timer will be started, although you will be allowed to continue eating if you are still hungry.”

The bowl of pasta will be refilled every 10 minutes to minimise any visual feedback in the regulation of appetite. To gain additional insight regarding psychological reward and food hedonics, a validated labelled magnitude scale[^23^](#_ENREF_23) will be administered following the first and last mouthful of this meal to reliably assess the degree of ‘pleasantness’ associated with ingestive behaviour. The 3 hour post-prandial period will then be repeated exactly as following breakfast (but without the 15-30 minute samples) to track metabolic responses to lunch.

***Objective ii –*** Phase II of testing will commence within 3-28 days after completion of each participants’ second trial under Phase I of testing (thus using participants’ breakfast trial along with the measures described above as a baseline). This trial schedule will again be used for all participants other than for eumenorrheic women, who will follow the same restrictions as described above and therefore occasionally be required to complete their first trial under Phase II prior to the completing the second trial under Phase I. In such cases, these participants’ 6-week intervention will simply be deferred for two weeks (i.e. enabling completion of Phase I) to allow follow-up measures to be taken at the same stage in the menstrual cycle. The first visit under Phase II will begin with participants again arriving in the laboratory at 8 am (±1 h) having adhered to the same standardisation procedures as described for Phase I. Measurements of body mass and adiposity based on hip and waist circumference (i.e. widest gluteal girth & mid-point between lowest rib and iliac crest, respectively), sagittal abdominal height (using a Holtain-Kahn calliper at the iliac crest) and body composition via DEXA (Hologic Discovery W) will also be made at this visit, before a small (~1 g) sample of subcutaneous adipose tissue will be acquired using a 14 G needle to determine basal expression of key genes related to appetite and physical activity regulation (e.g. adiponectin and leptin) and energy expenditure (e.g. UCPs).

This second phase of data collection will involve each participant being randomly assigned either to an extended daily fasting group (only plain water permitted until 1200 each day) or a breakfast consumption group (prescribed intake of ≥3000 kJ before 1100 each day, to include at least 1500 kJ within two hours of waking) for a period of 6 weeks [a duration previously shown to be sufficient for dietary modification to induce detectable changes in energy balance and body compositio^n.24^](#_ENREF_24) To facilitate this process, participants assigned to the breakfast group will be provided with detailed examples of appropriately energetic breakfasts and the energy content of other typical breakfast foods, although the breakfasts consumed will ultimately be self-selected by each individual on a daily basis. Upon completion of this 6 week dietary modification, participants will return to the laboratory for follow-up of all anthropometric measures and a second subcutaneous adipose tissue biopsy to determine whether the intervention has altered the expression of those genes measured at baseline (described above).

In relation to the stated objectives, this design therefore examines whether and how any acute alterations in energy balance can culminate in chronic changes in energy balance (i.e. the acute effects of each treatment may be modified as participants become accustomed and adapt to it). To further inform such questions, during the first and last week of the intervention participants will maintain detailed weighed records of their habitual food and fluid intakes for subsequent analysis of daily energy and macro-nutrient intakes using dietary analysis software (CompEat Pro). Additional data regarding feeding patterns will be gathered via analysis of time of day, daily frequency and energy content of individual eating occasions, with meals defined as ingestion of ≥1256 kJ at any given eating occasion and snacks defined as ingestion of <1256 kJ more than 45 minutes before or after a meal.[^25^](#_ENREF_25) All participants will receive telephone reminders to ensure appropriate compliance to the dietary record process, as has been advocated and applied to good effect by others.[^26^](#_ENREF_26) The food diaries provided to participants will also be accompanied by the same validated labelled magnitude scale[^23^](#_ENREF_23) that participants will have used during the first phase of testing to provide a rating of ‘pleasantness’ following the first and last mouthful of their lunchtime meal, thus informing research questions regarding psychological reward and food hedonics. Concurrent to these two periods of dietary recording, participants will also be fitted with a combined heart-rate/accelerometer (Actiheart, CamNtech) in order to accurately record energy expenditure/physical activity habits for the entire duration of each 7 day assessment period (which will also be complemented by daily records of participants physical in/activities and sleep/waking times). Importantly, at the point when this physical activity monitor is fitted, participants will be provided the following message both verbally and in writing to ensure that only genuinely meaningful behavioural responses are recorded: “Your lifestyle choices during this free-living monitoring period are central to this study. We are interested in any natural changes in your diet and/or physical activity habits, which you may or may not make in response to the intervention. This monitoring period has been carefully scheduled to avoid any pre-planned changes in these habits, such as a holiday or diet/exercise plan. You should inform us immediately if unforeseen factors external to the study may influence your lifestyle.”

After each participant’s follow-up trial under Phase II, participants will continue adhering to their assigned intervention for at least 2 days to allow for the 48 hour dietary control (although up to 7 days will be permitted in participants for whom either the menstrual cycle need not be controlled for or when both trials can still occur within the follicular phase) before returning to the laboratory to repeat their breakfast trial exactly as described in relation to Objective i. This trial will therefore inform whether acute metabolic and/or behavioural effects may be modified following chronic exposure to each treatment. A formal exit interview will also be incorporated into this final visit during the afternoon post-prandial period to obtain qualitative data regarding participants’ experiences of the study and motives for any perceived changes in behaviour.

***Objective iii –*** In relation to this objective, relevant data will be gathered during the second phase of testing to explore how interactions between extended daily fasting may relate to insulin resistance and cardiovascular disease risk (whether related to or independent of changes in energy balance). From a whole-body perspective, highly relevant data will be gathered during the first and last week of the 6 week intervention using a subcutaneous continuous glucose monitor (iPro, Medtronic) to record 24 hour glucose profiles for each participant (thus revealing whether either treatment alters either the average daily glucose concentration or the magnitude of hyperglycemic excursions following meals). This real-world indication of glycemic control will be complimented not only by simple comparison of changes in fasted glucose/insulin concentrations changes over the 6 weeks but also by way of an oral glucose tolerance test (OGTT) that will be conducted at baseline and follow-up in Phase II, immediately following each adipose tissue sample. At a more reductionist level, the adipose tissue samples will be subjected to further analysis to determine the sensitivity of this particular tissue to insulin. Specifically, on the day that these samples are collected, adipocytes will be isolated by collagenase digestion before determination of [U-^14^C]-D-glucose uptake at basal, submaximal (50 pmol·l^-1^) and maximal (20 nmol·l^-1^) insulin concentrations. This glucose uptake assay has been shown to accurately reflect 3-O-methylglucose transport under the conditions described and the resultant data will be expressed both as pmol·min^-1^ relative both to lipid mass[^31^](#_ENREF_31) and, following analysis of cell size, to cell surface area. This will be in addition to subsequent analyses for the total protein content of GLUT4 and Akt in this tissue and the expression of key genes implicated in lipolysis (e.g. HSL and ATGL), lipogenesis (e.g. PPARγ, PGC1a, SREBP1c) and more generally in glucose uptake and oxidation (e.g. GLUT4, PDK4, AMPKα-1/2, resistin, RBP4, leptin and adiponectin) and/or insulin signalling (e.g. IRS1/2, PI3K, Akt and TBC1 domain family member 4), thus informing whether there is any adaptive response to the intervention at the level of adipose tissue gene expression and protein content.

Finally, in relation to cardiovascular disease risk, measurements of blood lipid profiles, cytokine responses, CRP and blood pressure (DINAMAP Pro 100-400 V2, UK) will be contrasted between participants’ baseline and final follow-up trials, as will the expression of other relevant genes in adipose tissue samples (e.g. interleukin-6 and TNF-α). All hormones/cytokines to be measured in the proposed work will be quantified via ELISA, with whole blood glucose determined using a YSI analyser, plasma glucose, NEFA, cholesterol and urea measured using a spectrophotometer and gene expression using TaqMan® Real-Time PCR.

**Abbreviations**

Akt: also known as Protein Kinase B (PKB)

AMPK: Adenosine Monophosphate-Activated Protein Kinase

ATGL: Adipose Triglyceride Lipase

BMI: Body Mass Index

CCK: Cholecystokinin

CRP: C-Reactive Protein

DEXA: Dual Energy X-ray Absorptiometry

DIT: Diet induced Thermogenesis; also known as the Thermic Effect of Feeding (TEF)

ELISA: Enzyme Linked Immuno-Sorbent Assays

FMI: Fat Mass Index

GLUT: Glucose Transporter

HSL: Hormone Sensitive Lipase

IRS: Insulin Receptor Substrate

NEFA: Non-Esterified Fatty Acids

OGTT: Oral Glucose Tolerance Test

PCR: Polymerase Chain Reaction

PDK: Pyruvate Dehydrogenase Kinase

PGC: Peroxisome Proliferator-Activated Receptor Coactivator

PI3K: Phosphatidylinositol 3-kinase

PPAR: Peroxisome Proliferator-Activated Receptor

PYY: Peptide YY

RBP4: Retinol Binding Protein 4

RMR: Resting Metabolic Rate

SREBP: Sterol Regulatory Element-Binding Protein

TBC: Tre-2, Bub2p and Cdc16p

TNF-α : Tumor Necrosis Factor-α

UCP: Uncoupling Protein

**References**

1. Weinstein AR, Sesso HD, Lee IM, et al. The joint effects of physical activity and body mass index on coronary heart disease risk in women. Arch Intern Med 2008;168:884-90.

2. Ceriello A. Impaired glucose tolerance and cardiovascular disease: the possible role of post-prandial hyperglycemia. Am Heart J 2004;147:803-7.

3. Iqbal R, Anand S, Ounpuu S, et al. Dietary patterns and the risk of acute myocardial infarction in 52 countries: results of the INTERHEART study. Circulation 2008;118:1929-37.

4. Pearson TA, Mensah GA, Alexander RW, et al. Markers of inflammation and cardiovascular disease: application to clinical and public health practice. Circulation 2003;107:499-511.

5. Aronson D, Bartha P, Zinder O, et al. Association between fasting glucose and C-reactive protein in middle-aged subjects. Diabet Med 2004;21:39-44.

6. Kang ES, Kim HJ, Kim YM, et al. Serum high sensitivity C-reactive protein is associated with carotid intima-media thickness in type 2 diabetes. Diabetes Res Clin Pract 2004;66:S115-20.

7. Rutter MK, Meigs JB, Sullivan LM, D'Agostino RB, Sr., Wilson PW. C-reactive protein, the metabolic syndrome, and prediction of cardiovascular events in the Framingham Offspring Study. Circulation 2004;110:380-5.

8. Schulz KF, Altman DG, Moher D. CONSORT 2010 Statement: updated guidelines for reporting parallel group randomised trials. Trials 2010;11:32.

9. Kelly TL, Wilson KE, Heymsfield SB. Dual energy X-Ray absorptiometry body composition reference values from NHANES. PLoS ONE 2009;4:e7038.

10. Schulz KF, Grimes DA. Unequal group sizes in randomised trials: guarding against guessing. Lancet 2002;359:966-70.

11. Pottgiesser T, Specker W, Umhau M, Dickhuth HH, Roecker K, Schumacher YO. Recovery of hemoglobin mass after blood donation. Transfusion (Paris) 2008;48:1390-7.

12. Biswas A, Viegas OA, Coeling Bennink HJ, Korver T, Ratnam SS. Implanon contraceptive implants: effects on carbohydrate metabolism. Contraception 2001;63:137-41.

13. Verboeket-van de Venne WP, Westerterp KR, Kester AD. Effect of the pattern of food intake on human energy metabolism. Br J Nutr 1993;70:103-15.

14. Schlundt DG, Hill JO, Sbrocco T, Pope-Cordle J, Sharp T. The role of breakfast in the treatment of obesity: a randomized clinical trial. Am J Clin Nutr 1992;55:645-51.

15. Schulz KF, Grimes DA. Sample size slippages in randomised trials: exclusions and the lost and wayward. Lancet 2002;359:781-5.

16. Buffenstein R, Poppitt SD, McDevitt RM, Prentice AM. Food intake and the menstrual cycle: a retrospective analysis, with implications for appetite research. Physiol Behav 1995;58:1067-77.

17. Lissner L, Stevens J, Levitsky DA, Rasmussen KM, Strupp BJ. Variation in energy intake during the menstrual cycle: implications for food-intake research. Am J Clin Nutr 1988;48:956-62.

18. Solomon SJ, Kurzer MS, Calloway DH. Menstrual cycle and basal metabolic rate in women. Am J Clin Nutr 1982;36:611-6.

19. Compher C, Frankenfield D, Keim N, Roth-Yousey L. Best practice methods to apply to measurement of resting metabolic rate in adults: a systematic review. J Am Diet Assoc 2006;106:881-903.

20. Stubbs RJ, Hughes DA, Johnstone AM, et al. The use of visual analogue scales to assess motivation to eat in human subjects: a review of their reliability and validity with an evaluation of new hand-held computerized systems for temporal tracking of appetite ratings. Br J Nutr 2000;84:405-15.

21. Chryssanthopoulos C, Williams C, Nowitz A, Bogdanis G. Skeletal muscle glycogen concentration and metabolic responses following a high glycaemic carbohydrate breakfast. J Sports Sci 2004;22:1065-71.

22. Manning PJ, Sutherland WH, McGrath MM, de Jong SA, Walker RJ, Williams MJ. Postprandial cytokine concentrations and meal composition in obese and lean women. Obesity 2008;16:2046-52.

23. Haase L, Cerf-Ducastel B, Buracas G, Murphy C. On-line psychophysical data acquisition and event-related fMRI protocol optimized for the investigation of brain activation in response to gustatory stimuli. J Neurosci Methods 2007;159:98-107.

24. Keim NL, Van Loan MD, Horn WF, Barbieri TF, Mayclin PL. Weight loss is greater with consumption of large morning meals and fat-free mass is preserved with large evening meals in women on a controlled weight reduction regimen. J Nutr 1997;127:75-82.

25. de Castro JM. Accommodation of particular foods or beverages into spontaneously ingested evening meals. Appetite 1994;23:57-66.

26. Dreon DM, Frey-Hewitt B, Ellsworth N, Williams PT, Terry RB, Wood PD. Dietary fat:carbohydrate ratio and obesity in middle-aged men. Am J Clin Nutr 1988;47:995-1000.

27. Corder K, Brage S, Mattocks C, et al. Comparison of two methods to assess PAEE during six activities in children. Med Sci Sports Exerc 2007;39:2180-8.

28. Thompson D, Batterham AM, Bock S, Robson C, Stokes K. Assessment of low-to-moderate intensity physical activity thermogenesis in young adults using synchronized heart rate and accelerometry with branched-equation modeling. J Nutr 2006;136:1037-42.

29. Foley JE, Kashiwagi A, Verso MA, Reaven G, Andrews J. Improvement in in vitro insulin action after one month of insulin therapy in obese noninsulin-dependent diabetics. Measurements of glucose transport and metabolism, insulin binding, and lipolysis in isolated adipocytes. J Clin Invest 1983;72:1901-9.

30. Kashiwagi A, Verso MA, Andrews J, Vasquez B, Reaven G, Foley JE. In vitro insulin resistance of human adipocytes isolated from subjects with noninsulin-dependent diabetes mellitus. J Clin Invest 1983;72:1246-54.

31. Liu SC, Wang Q, Lienhard GE, Keller SR. Insulin receptor substrate 3 is not essential for growth or glucose homeostasis. J Biol Chem 1999;274:18093-9.
